# Long-term kinetics of proviral load in HTLV-1 carriers: defining risk for the development of adult T-cell leukemia/lymphoma

**DOI:** 10.1186/s40364-025-00747-5

**Published:** 2025-02-26

**Authors:** Koji Jimbo, Masanori Nojima, Keiko Toriuchi, Makoto Yamagishi, Makoto Nakashima, Yoshihisa Yamano, Kaoru Uchimaru, Yasuhito Nannya

**Affiliations:** 1 Division of Hematopoietic Disease Control, The Institute of Medical Science, The University of Tokyo, 4-6-1 Shirokanedai, Minato-Ku, Tokyo 108-8639 Japan; 2 Department of Hematology/Oncology, Research Hospital, The Institute of Medical Science, The University of Tokyo, Tokyo, Japan; 3 Center for Translational Research, Research Hospital, The Institute of Medical Science, The University of Tokyo, Tokyo, Japan; 4 Laboratory of Tumor Cell Biology, Department of Computational Biology and Medical Sciences, Graduate School of Frontier Sciences, The University of Tokyo, Tokyo, Japan; 5 Laboratory of Viral Oncology and Genomics, Department of Computational Biology and Medical Sciences, Graduate School of Frontier Sciences, The University of Tokyo, Tokyo, Japan; 6 Department of Rare Diseases Research, Institute of Medical Science, St. Marianna University School of Medicine, Kanagawa, Japan; 7 Department of Neurology, St. Marianna University School of Medicine, Kanagawa, Japan

**Keywords:** Human T-lymphotropic virus 1 (HTLV-1), Adult T-cell leukemia/lymphoma (ATL), Proviral load, Trajectory analysis, Risk estimation

## Abstract

**Background:**

Assessment of adult T-cell leukemia/lymphoma (ATL) development among human T-lymphotropic virus 1 (HTLV-1)-infected individuals (carriers) constitute a significant issue. A high HTLV-1 proviral load (PVL) in carriers has been used as a risk factor for ATL development and PVLs are considered to remain unchanged over time among carriers.

**Methods:**

This single-center analysis used a cohort from a prospective observational study of HTLV-1 carriers in Japan (JSPFAD). Carriers whose PVL was measured at least twice between October 2004 and March 2023 were included. We used trajectory analysis to construct a kinetic model of the PVL.

**Results:**

Analysis of 1371 samples from 252 carriers revealed a slight but significant increase in the PVL with age (*P* < 0.001). Trajectory analysis of PVL kinetics classified the carriers into six groups, in three of which increased over time. When we applied the model to 15 carriers who subsequently developed ATL, 12 (80%) were classified into the highest PVL group, with an estimated 15-year ATL development of 47.5% (95% confidence interval: 20.4–74.2%). Notably, younger patients are at greater risk of developing ATL if their PVL values are comparable. Our risk estimation model is available online (https://atlriskpredictor.shinyapps.io/ATL_risk_calculator/).

**Conclusions:**

This study demonstrated that the PVLs increases over time, allowing for prospective risk estimation for ATL development. Further validation is needed to assess the validity of this model.

**Trial registration:**

Retrospectively registered.

**Supplementary Information:**

The online version contains supplementary material available at 10.1186/s40364-025-00747-5.

## Background

Human T-lymphotropic virus 1 (HTLV-1) infection is endemic in some areas, including southwestern Japan, sub-Saharan Africa, South America, the Caribbean, and foci in the Middle East and central Australia [1]. Individuals who have HTLV-1 usually exhibit no symptoms and are referred to as asymptomatic carriers; however, they are at risk of developing various HTLV-1-related diseases, such as adult T-cell leukemia/lymphoma (ATL), HTLV-1–associated myelopathy (HAM), and HTLV-1–associated uveitis (HU) [2–7]. ATL is a disease in which HTLV-1-infected cells acquire clonality and oncogenicity [2–5]. ATL is a refractory T-cell lymphoma with a poor prognosis, which occurs in approximately 5% of HTLV-1 carriers at some point in their lifetime [8–11]. Since ATL is a fatal disease, the ability to make predictions regarding its development is an important aspect of follow-up programs for carriers of HTLV-1.

HTLV-1 is a retrovirus that infects CD4 + T cells and integrates itself as a provirus into host genomic DNA through reverse transcription of the viral RNA [12–14]. HTLV-1 increases the proviral load (PVL) through cell-to-cell transmission from infected to uninfected cells and the proliferation of infected cells [13–16]. During the clinical follow-up of HTLV-1 carriers, PVL is a useful proxy for the quantification of HTLV-1-infected cells. PVLs are higher in patients with HTLV-1-related diseases compared with HTLV-1 carriers and have been shown to be an indicator of disease progression [17–22]. Therefore, PVL may be useful for predicting the development of HTLV-1-related diseases. In particular, high PVLs represent a risk factor for the development of ATL in HTLV-1 carriers, independent of other risk factors, including advanced age, a family history of ATL, and an incidental diagnosis of HTLV-1 infection during the treatment of other diseases [21].

Although the dynamics of PVL during the early phase of HTLV-1 infection remain unexplored, it is assumed that PVLs increase steeply during the early phase of infection before reaching a steady state in the subsequent phase [23]. There have been several long-term follow-up studies, of up to approximately 10 years, of PVLs in carriers of HTLV-1, but none has clearly demonstrated an increase in PVLs over time [17, 18, 24–26], supporting the notion that PVLs enter a steady-state phase. While PVLs increase when HTLV-1-infected cells acquire clonality and ATL develops, it is assumed that PVLs remain largely unchanged during the HTLV-1 carrier period. However, the long-term dynamics of PVLs over a period of 10 years or more remain a matter of speculation.

Assuming the currently held view that PVLs do not change greatly over time is correct, it follows that the impact of PVLs on the development of ATL does not change greatly with age. However, to test the validity of this hypothesis, it is necessary to analyze PVL data over a long period.

In this study, we first attempted to construct a long-term kinetic model of PVLs through the mathematical analysis of PVL data from HTLV-1 carriers. We then used this model to estimate the risk of developing ATL, which depends on PVL, more accurately than has been achieved previously.

## Methods

### Patients and samples

We enrolled HTLV-1 carriers according to the following inclusion criteria. They had visited the Research Hospital, the Institute of Medical Science, The University of Tokyo, between October 2004 and March 2023; they agreed to participate in the Joint Study on Predisposing Factors of ATL Development (JSPFAD); and they had their PVLs evaluated at least twice. JSPFAD is a nationwide, multicenter prospective study of HTLV-1 carriers in Japan. The participants provided written informed consent to participate in JSPFAD, in accordance with the principles set out in the Declaration of Helsinki. This study was approved by the Institutional Review Board of the Institute of Medical Science, The University of Tokyo (2019–72–0217; 2022–69–0203). PVLs were measured via real-time polymerase chain reaction (PCR) using the ABI PRISM 7000 Sequence Detection System and Applied Biosystems 7500 Real-time PCR system (both from Applied Biosystems Japan) as previously described [21, 27, 28] with minor modifications. DNA extraction from cells was performed using a QIAamp DNA Blood Mini Kit (QIAGEN, Hilden, Germany). PVL was measured by real-time PCR of the pX region of the HTLV-1 virus and corrected by the human gene encoding the RNase P enzyme. The primers and probes used in this study are as follows: forward primer pX2-S 5’-CGGATACCCAGTCTACGTGTT-3’; reverse primer pX2-AS 5’-CAGTAGGGCGTGACGATGTA-3’; carboxyfluorescein (FAM)-labeled pX2 probe 5’-CTGTGTACAAGGCGACTGGTGCC-3’; and TaqMan RNase P Control Reagents (VIC dye) (Applied Biosystems, #4,316,844). PVLs are reported as copies per 100 peripheral blood mononuclear cells (PBMCs). Other clinical information was obtained from participants’ medical records. For the estimation of PVL kinetics in HTLV-1 carriers, we excluded patients who developed ATL; we then constructed the model. To estimate the risk of HTLV-1 carriers developing ATL, we applied the age and PVL data of HTLV-1 carriers who later developed ATL to the model.

### Statistical analysis

The Tukey–Kramer multiple comparison test was used to compare continuous variables by age groups, and the Jonckheere-Terpstra test was used to test for monotonically increasing trends. Logarithmic transformation was applied to PVLs due to their right-skewed distribution. The trajectory analysis was then performed using a latent class mixed model (LCMM) to model longitudinal changes in PVLs. This approach allowed us to capture heterogeneity within the population by identifying distinct latent classes that exhibited different trajectories over time. The choice of the number of latent classes was guided by model fit indices, such as the Akaike information criterion (AIC). The most efficient model was selected based on the AIC from models showing relationship between linear, quadratic, or cubic terms and age. A linear regression model was applied to test for linearity and to estimate PVLs at specific time points, as well as the doubling times of PVLs before and after the identification of latent classes. Kaplan–Meier analysis was performed as a time-to-event analysis for ATL development. *P-*values of < 0.05 were considered to be statistically significant. All the statistical analyses were performed using R (version 4.3.1; R Foundation for Statistical Computing) or GraphPad Prism software (version 7.0d; GraphPad Software). The R package “lcmm” was used for the trajectory analysis.

### Publication of our model online

We constructed an online application that implements our model for predicting the risk of developing ATL based on single- or multi-point PVLs and age (https://atlriskpredictor.shinyapps.io/ATL_risk_calculator/) .This application returns the estimated risk group and Kaplan–Meier-based estimate of the risk of developing ATL.

## Results

### Relationship between age and PVL

**Fig. 1:**
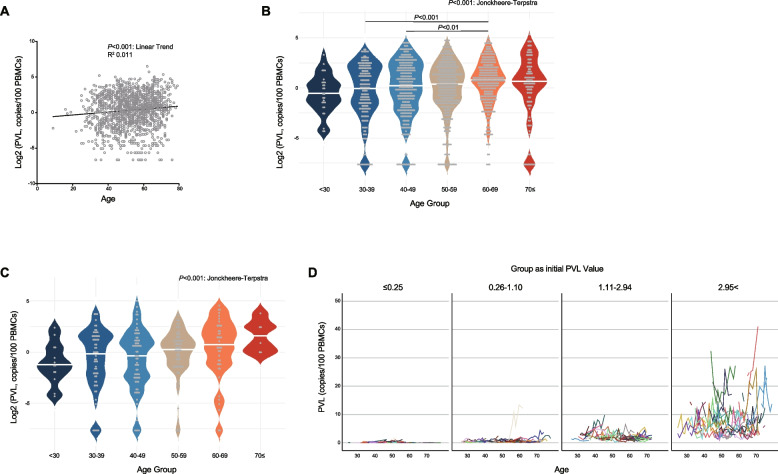
Relationship between age and PVL. **A** Correlation between age and log-transformed PVL (n = 1371). **B** Violin plots showing PVLs of all samples (n = 1371) for each age group. **C** Violin plots showing PVLs of the first sample from each HTLV-1 carrier (n = 252) in each age group. **D** Rate of change in PVLs in the four groups categorized by their initial PVL values. *P*-values were calculated using the Spearman rank correlation (**A**), Tukey–Kramer multiple test (**B**), or Jonckheere-Terpstra test (**B**, **C**)

We analyzed the PVL kinetics of HTLV-1 carriers in 252 cases of 1371 specimens, excluding 15 cases who developed ATL out of 267 HTLV-1 carriers in the entire cohort (Table 1). Sixty-nine cases (27%) were male, with a median of four samples per case (range 2–26) and a median observation period of 5 years (range 1–18). The median age of the sample was 53 years (range 16–79), and the median PVL was 1.40 copies/100 PBMCs (interquartile range, IQR 0.37–4.11). We then evaluated the correlation between age and PVL. The log-transformed PVLs (log(PVLs)) showed a significant linear correlation with age (Fig. 1A). When PVL was evaluated by age group, individuals in their 60s had significantly higher PVLs than those in their 30s and 40s, and a trend test revealed a positive correlation between PVL and age group (Jonckheere-Terpstra analysis: *P* < 0.001) (Fig. 1B). A similar trend was observed when restricted to the first specimen from each HTLV-1 carrier (Fig. 1C). The soluble interleukin-2 receptor (sIL-2R) level was not clearly correlated with age, but the percentage of abnormal lymphocytes in the peripheral blood increased with age (Figure S1A-D). To examine PVL dynamics over time in individuals after they had been diagnosed as HTLV-1 carriers, we investigated changes in PVLs from the initial PVL in patients whose PVL was measured at least three times. We classified the HTLV-1 carriers into four groups, using PVL IQR values of 0.25, 1.10, and 2.94 as cutoffs, and found that the larger the initial PVL, the greater the rate of change in PVL (Fig. 1D). These results suggest that PVLs increase with age, as the overall trend and the degree of changes depend on the initial value of PVL.

**Table 1:** Characteristics of HTLV-1 carriers without development of ATL, with risk classification and trajectory group

**Risk Classification**	**High**	**High-Int**	**Low-Int**	**Low**			**Total**
**Trajectory Group**	**A**	**B**	**C**	**D**	**E**	**F**	
**Number of Cases**	32 (13%)	55 (22%)	53 (21%)	61 (24%)	29 (12%)	22 (9%)	252
**Sex, Male (%)**	11 (34%)	16 (29%)	16 (30%)	15 (25%)	6 (21%)	5 (23%)	69 (27%)
**Median Number of Samples per Patients (range)**	6 (2–26)	4 (2–16)	4 (2–15)	4 (2–15)	4 (2–11)	3.5 (2–13)	4 (2–26)
**Median Follow-up Time, years (range)**	6.5 (1–18)	6 (1–16)	5 (1–15)	5 (1–16)	4 (1–16)	4 (1–13)	5 (1–18)
**Samples**	228 (17%)	307 (22%)	297 (22%)	329 (24%)	116 (8%)	94 (7%)	1371
**Age Distribution (%)**							
** < 20**	0 (0%)	3 (1%)	0 (0%)	1 (0.3%)	0 (0%)	0 (0%)	3 (0.2%)
** 20–29**	6 (3%)	0 (0%)	3 (1%)	10 (3%)	5 (4%)	1 (1%)	25 (1.8%)
** 30–39**	51 (22%)	33 (11%)	22 (7%)	43 (13%)	22 (19%)	19 (20%)	190 (14%)
** 40–49**	73 (32%)	62 (20%)	54 (18%)	70 (21%)	47 (42%)	21 (22%)	328 (24%)
** 50–59**	46 (20%)	107 (35%)	77 (26%)	103 (31%)	22 (19%)	31 (33%)	386 (28%)
** 60–69**	38 (17%)	84 (27%)	106 (36%)	84 (26%)	13 (11%)	10 (11%)	335 (24%)
** ≥ 70**	14 (6%)	18 (6%)	35 (11%)	18 (5%)	7 (6%)	12 (13%)	104 (7.6%)
**Sex, Male (%)**	92 (40%)	99 (32%)	82 (27%)	92 (28%)	36 (31%)	24 (26%)	425 (31%)
**Median Value of PVL, copies/100 PBMCs (IQR)**	9.07 (6.13–14.05)	3.65 (2.52–5.16)	1.46 (1.08–1.93)	0.43 (0.28–0.62)	0.095 (0.050–0.150)	0.00 (0.00–0.02)	1.40 (0.37–4.11)
**PVL at 20-year Old, copies/100 PBMCs (IQR)**	4.18 (3.53–4.94)	1.57 (1.31–1.88)	0.74 (0.61–0.89)	0.39 (0.32–0.48)	0.16 (0.12–0.22)	0.01 (0.01–0.02)	0.59 (0.44–0.79)
**PVL Doubling Time, year (IQR)**	25.5 (21.3–31.3)	28.2 (23.3–35.7)	39.8 (31.3–55.6)	405.5 (90.9-Infinite)	NA	701.3 (33.3-Infinite)	41.7 (27.8–90.9)
**R**^**2**^** for age and log-transformed PVL** **(*P*-value)**	0.312 (*P* < 0.001)	0.232 (*P* < 0.001)	0.141 (*P* < 0.001)	0.001 (*P* = 0.571)	0.108 (*P* < 0.001)	0.000 (*P* = 0.920)	0.011 (*P* < 0.001)
				0.001 (*P* = 0.406; when combined groups D-F)			
**Median Value of sIL-2R, U/mL (IQR)**	432.5 (364.0–549.0)	366.0 (298.5–464.0)	356.0 (277.0–451.0)	293.0 (246.0–357.0)	285.0 (235.0–349.0)	287.0 (242.8–381.0)	340.0 (272.0–435.0)
**Median Value of abnormal lymphocytes, % (IQR)**	2.0 (1–3.2)	1.0 (0.5–1.5)	0.5 (0.3–1.0)	0.5 (0–1.0)	0.5 (0–0.5)	0.5 (0–1.0)	0.5 (0.4–1.5)

*Int* intermediate, *PVL* proviral load, *sIL-2R* soluble interleukin-2 receptor, *IQR* interquartile range

### Mathematical model for long-term kinetics of PVL in carriers of HTLV-1

Based on the results described above, we hypothesized that PVL dynamics could be divided into several groups. We applied trajectory analysis to evaluate this hypothesis. The conditions for setting up the model were as follows: 1) changes in PVLs were divided into classes, with different initial values and time rates of change, and the probability of belonging to each class was calculated; 2) the class to which a patient belonged did not change during the course of serial sample evaluation. First, the dimension of age in the regression model was determined based on the AIC (Figure S2A). This finding is compatible with the linear correlation between log(PVL) and age (Fig. 1A). The number of latent classes (K) was determined based on the improvement in the AIC when incrementing the K value. As the AIC improvement rate between K and K-1 was > 5% for K-values ≤ 6, and < 5% for K-values > 6, we determined that K = 6 was the appropriate number of latent classes (Figure S2B). These classes were designated A to F according to the intercept of the regression curves.

**Fig. 2:**
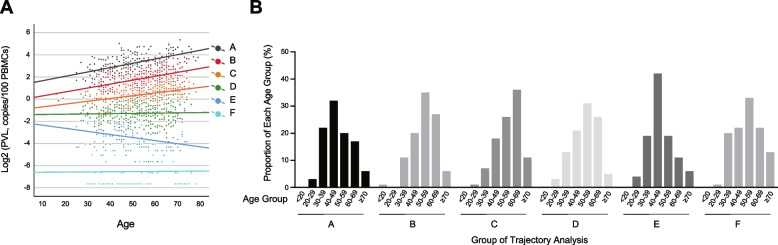
Grouping of PVL kinetics by trajectory analysis. **A** Correlation between age and log-transformed PVL grouped by trajectory analysis. The dots are color-coded according to the groups (A to F, corresponding to high to low PVL groups) identified by the trajectory analysis. **B** Age distribution among the six groups identified by trajectory analysis

The three groups with high initial PVL values presented a linear increase in log(PVL) with age, whereas the remaining three groups presented no significant increase or decrease in PVLs with age (Table 1, Fig. 2A). Among the three groups that presented an increase in PVLs (groups A-C), the group with high initial PVLs also had a greater change in PVLs than those with low PVLs (Table 1). In addition, the distribution of ages in these groups was not highly skewed (Fig. 2B). These results indicate that PVL kinetics can be divided into six groups based on PVL and age, with three groups showing an increase in PVL with age and the other three groups exhibiting no significant change.

### Evaluation of the risk of developing ATL using our model of PVL kinetics

**Fig. 3:**
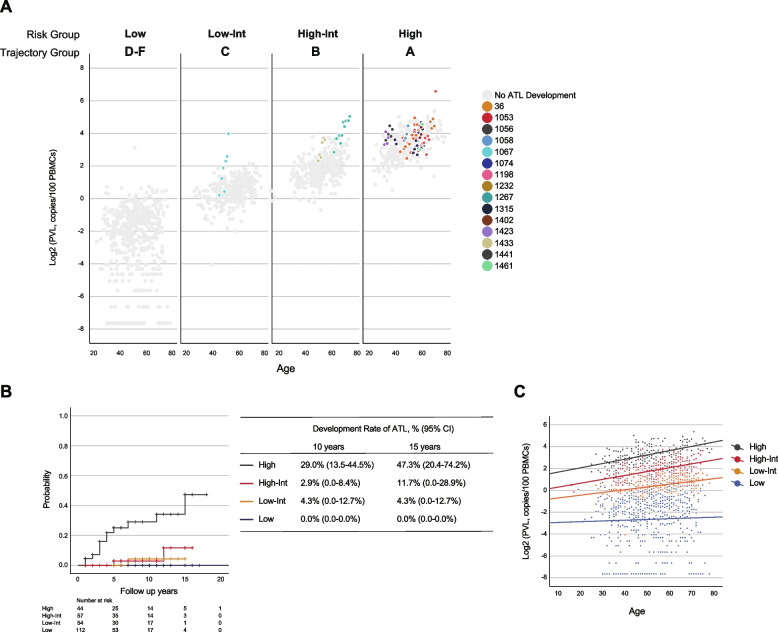
Using the PVL kinetics model to estimate the risk of developing ATL. **A** Log-transformed PVL transition of 15 patients who were HTLV-1 carriers and went on to develop ATL according to the 4-risk groups (High, High-Int, Low-Int, and Low) determined by trajectory analysis and risk of ATL development. **B** Cumulative incidence of ATL development according to the 4-risk groups for ATL development. **C** Correlation between age and log-transformed PVL according to the 4-risk groups for ATL development

To estimate the risk of developing ATL, 15 cases who developed ATL during the follow-up of HTLV-1 carriers were applied to the model of PVL kinetics (Table 2). In the overall cohort of 267 cases, the incidence of ATL development was 5.6%. The median age of ATL development was 60 years (range 36–76). All patients had smoldering-type ATL at development, but six patients progressed to aggressive ATL that required treatment and one patient progressed to chronic-type ATL. First, to investigate whether a single PVL value could be used to estimate the risk of developing ATL, we applied the first measurement alone to estimate the risk. Twelve patients (80%) were classified as A, two patients (13%) as B, and one patient (7%) as C (Table 2, Fig. 3A). These groups were designated as high-risk (“High”), high-to-intermediate-risk (“High-Int”), and low-to-intermediate-risk (“Low-Int”) groups for the development of ATL, respectively. As there were no developed ATL patients in the three groups with low PVL and no increase over time, they were combined into the low-risk (“Low”) group. In the High group, the 10-year incidence of ATL was 29% (95% confidence interval, CI, 13.5–44.5), and the 15-year incidence was 47.3% (95% CI, 20.4–74.2) (Fig. 3B). While the High and High-Int groups had similar slopes for the regression curves, the intercepts were very different (Table 1, Fig. 3C). These results suggest that our PVL kinetics model can be used to predict the risk that carriers of HTLV-1 will go on to develop ATL.

**Table 2:** Characteristics of HTLV-1 carriers with later development of ATL

**HTLV-1 carriers**	***n* = 15**
Sex, Male (%)	7 (47%)
Median Number of Samples per Patients (range)	6 (2–16)
Median Follow-up Time, years (range)	4 (1–15)
Trajectory Group / Risk Classification	
A / High Risk	12 (80%)
B / High-Int Risk	2 (13%)
C / Low-Int Risk	1 (7%)
PVL ≥ 4% at First Sample	14 (93%)
Median Age at ATL Development (range)	60 (36–76)
Type of ATL at Development: Smoldering (%)	15 (100%)
Development to Aggressive ATL Later	6 (40%)
**Samples**	***n* = 94**
Median Age of the Samples (range)	57 (33–76)
Age Distribution (%)	
< 30	0 (0%)
30–39	9 (10%)
40–49	11 (12%)
50–59	37 (39%)
60–69	28 (30%)
≥ 70	9 (10%)
Sex, Male (%)	47 (50%)
Median Value of PVL, copies/100 PBMCs (IQR)	12.8 (9.3–18.8)
Median Value of sIL-2R, U/mL (IQR)	430.5 (344.3–588.8)
Median Value of Abnormal Lymphocytes, % (IQR)	3.0 (2.0–5.0)

*ATL* adult T-cell leukemia/lymphoma, *PBMSc* peripheral blood mononuclear cells, *PVL* proviral load, *sIL-2R* soluble interleukin-2 receptor, *IQR* interquartile range

It has been reported that a PVL of 4 copies/100 PBMCs or more is associated with a high risk of developing ATL [21], and this value has been used in clinical practice to estimate the risk of developing ATL. Therefore, we examined whether our prediction system could further stratify patients conventionally defined as being at high-risk (PVL ≥ 4 copies/100 PBMCs). Sixty-one patients had initial PVLs ≥ 4 copies/100 PBMCs, and 40 and 21 patients were classified as being High and High-int risk, respectively (Figure S3). The incidence of ATL development in the High group of patients was 32.8% (95% CI, 15.6–50.0%) at 10 years and 50.4% (95% CI, 21.4–76.7%) at 15 years, compared with 7.3% (95% CI, 0.0–20.6%) and 22.6% (7.1–52.4%) in the High-Int group, at 10 and 15 years, respectively (Figure S3). Our results indicate that our model provides a more precise prediction of the development of ATL among those conventionally considered to be high-risk who have a PVL of ≥ 4 copies/100 PBMCs.

Given that PVL increases with age in groups with high PVL, we then evaluated whether young cases are at greater risk of developing ATL than old cases at similar PVL values. When we focused on 31 patients with a PVL of 4–8 copies/100 PBMCs at the initial measurement who were on the borderline between the High and High-Int groups, 10 (53%) of 19 cases aged ≤ 55 years were classified as High and 3 (16%) actually developed ATL, whereas just 1 of 12 cases aged > 55 years (8.3%) was in the High group and developed ATL (Table S1). This result supports the notion that younger patients should be classified as higher risk and have a higher incidence of ATL among those with similar PVL values. Therefore, a risk assessment should be performed, taking the age at which PVL was measured into account.

We also examined whether there was any benefit to applying multi-point measurements to our model. To this point, we used only initial PVL measurements to predict the risk of developing ATL. When we applied the first five measurements for each patient, two cases were reclassified from the High-Int to the High group. When we applied six or more measurements, one case was reclassified from the Low-Int to the High-Int group (Table S2). All the patients classified in the High group based on the initial measurement alone remained in this group even when the number of measurement points was increased. The fact that multi-point measurements classified individuals with ATL development at higher risk group compared to single-point measurements suggests that multi-point measurements allowed more accurate assignment of each case to risk classes. Therefore, multi-point application is desirable for predicting ATL development, but additional measurements require routine follow-up programs and additional costs.

Our results suggest that our model is a useful tool for more accurately estimating the risk of developing ATL development and in a more granular manner than was previously possible.

### Prospective risk estimation for the development of ATL based on age and PVL

**Fig. 4:**
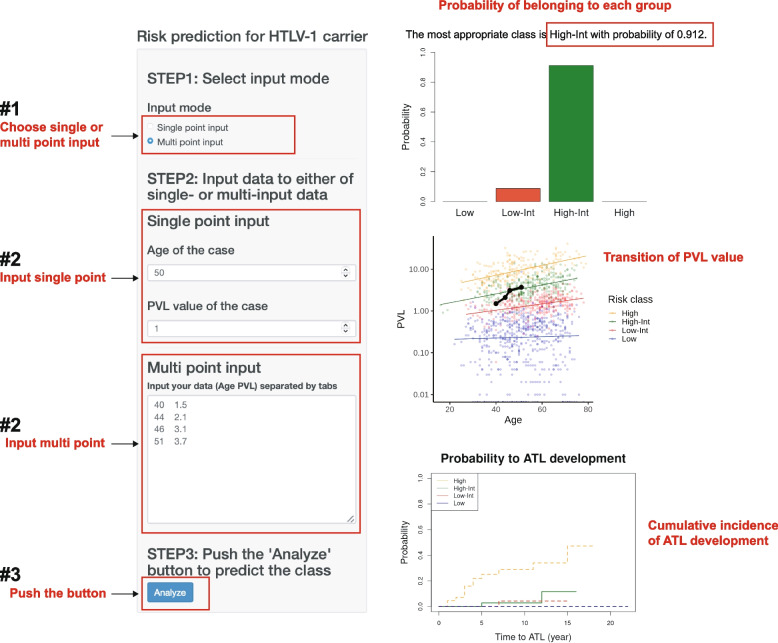
Web-based application for estimating the risk of developing ATL. By entering the single- or multi-point PVL and age, the probability of belonging to a particular group in the model is output. The PVL trends for each group and the cumulative incidence of developing ATL for each group are also displayed

By making it possible to estimate which risk group a patient belongs to, using our PVL kinetic model and based on the two variables of age and PVL, we can now estimate the risk of developing ATL more precisely than was possible before. We have created a tool that uses a Shiny application to estimate which group a patient belongs to and have made this available online (Fig. 4, https://atlriskpredictor.shinyapps.io/ATL_risk_calculator/). By entering a patient’s PVL and their age at the time of measurement, the probability of belonging to any group in the model is generated as outputs. In addition, the PVL trends for each group and the probability of developing ATL for each group defined from this model are displayed. Both single- and multi-point inputs are possible, with multi-point inputs enabling more accurate grouping. This tool makes it possible to prospectively classify the risk that a carrier of HTLV-1 will develop ATL.

## Discussion

To the best of our knowledge, this is the first report to describe long-term PVL kinetics in carriers of HTLV-1. The PVL kinetic model we developed is useful for estimating the risk of ATL development in HTLV-1 carriers.

We showed that PVL was correlated with age for our cohort overall and that PVL trends could be categorized into six groups, some of which showed increasing PVL with age. Although some studies have followed individuals who carry HTLV-1 for periods of up to approximately 10 years, no trends showing increasing PVL have been observed [17, 18, 24–26]. There are two reasons why we were able to reveal this increasing trend in PVL. First, our PVL kinetics model allowed us to simulate a 50-year observation period. Second, our model revealed that PVL increased in some groups but not others, with the trend of increasing PVL for the cohort as a whole being very small. In fact, the PVL doubling time was 25.5 years even in the group with the largest increasing trend, which may explain why it is difficult to observe a clear increasing trend without grouping patients. A model of clonal evolution is envisioned in which, during the HTLV-1 carrier stage, myriad HTLV-1-infected cell clones are present in the peripheral blood, and clonal selection gradually leads to tumorigenesis and the expansion of specific clones, resulting in the development of ATL [29, 30]. Considering the results of our study, it can be assumed that the clonal evolution of HTLV-1-infected cells over decades results in a gradually increasing trend in PVL values. This view is supported by the fact that ATL incidence actually occurs only in the groups that show an increasing trend in PVL.

It is also necessary to consider the possibility that the three groups with low initial PVLs and no increasing trend (trajectory group D-F) had very low absolute PVLs, which may have resulted in less accurate quantification. Furthermore, we cannot exclude the possibility that an extremely gradual increasing trend may occur in the low PVL group over a long period of time and that this is difficult to observe. However, this is a negligible transition, at least in actual clinical practice considering the life expectancy of the individual. Since a detectable increase in PVL was actually observed only in the Low-Int or higher group in this model, it is necessary and sufficient to take into account the increase trend of PVL when a case is classified into the Low-Int or higher group.

Factors that promote the development of ATL include internal factors, such as HTLV-1 viral gene mutations and changes in virus-derived protein expression, and external factors, such as the immune environment and host genes [31–35]. It is assumed that the specific combination of these factors, in addition to other, unknown factors, will define the rate at which ATL develops. As these factors can alter the PVL, it may serve as a conveniently observable parameter that acts as a quantifiable measure of the sum of these factors. Therefore, it is not difficult to imagine that PVL is a risk factor for the development of ATL; indeed, it is already recognized that a PVL of 4 copies or more per 100 PBMCs is considered to be a risk factor for the development of ATL [21]. Our PVL kinetic model further stratified the risk in the group with a PVL of 4 copies or more per 100 PBMCs and revealed that younger carriers have a higher risk of ATL development than older carriers who have similar PVLs. These results suggest that our model, which takes age into account in addition to PVL, is useful for the accurate and granular prediction of ATL development. But it should be noted that PVL is a quantified indicator of the amount of virus present in T lymphocytes and can therefore fluctuate depending on comorbid conditions such as infection and inflammation. Therefore, PVL measurements in the absence of such comorbidities or at multiple time points are desirable.

It is also important to note that we should pay attention to the cases with low PVL at a young age. Both the High-Int and Low-Int groups in this study had PVL < 4 copies/100 PBMCs at age 20, but some patients developed ATL in both groups. This suggests that a subset of the patient population who may develop ATL may have a low PVL at younger ages. In addition, the fact that the patients who developed ATL were reclassified into a higher-risk group by applying a multiple-point sample rather than a single-point sample also suggests that HTLV-1 carriers who develop ATL tend to show an increase in PVL over time, whereas HTLV-1 carriers who do not develop ATL do not show this trend. Actually, the carriers who developed ATL showed a trend toward increased PVL, even in cases with initially low PVL (Fig. 3A). Rapid clonal evolution of HTLV-1-infected cells due to the acquisition of gene mutations that promote tumorigenesis has also been reported [36, 37], but repeated measurements of PVL can predict this clonal expansion. Although the PVL increase in the cohort as a whole was slight and did not show a significant increasing trend in the group with low initial PVL, it is important to measure PVL repeatedly on an annual basis who are classified as being Low-Int or higher at a single measurement, since those who develop ATL may show a trend toward increased PVL.

This study had several limitations. First, it was a single-center, retrospective study conducted in Japan only. Geographic factors should also be considered in the impact of the follow-up of HTLV-1 carriers [38], so a larger, worldwide prospective study is desirable. Second, the number of carriers who went on to develop ATL was small (15 patients). Although the rarity of the disease makes it difficult to include a large number of HTLV-1 carriers who develop ATL, analysis of a large number of cases is warranted to ensure the safety of individuals who are classified in the Low group. In our study, no patients in the Low group developed ATL. Third, in this study we did not obtain any information regarding factors other than PVL and age, such as clonality and the presence of mutated genes. It would be prudent to estimate the risk of developing ATL when taking these other factors into consideration. Fourth, the route of infection has not been identified in many cases. It is possible that PVL kinetics differ between horizontally and vertically infected cases, and future verification is desirable. Finally, it should be noted that there are no patients in this cohort who developed lymphoma-type ATL. It has been suggested that some cases of lymphoma-type ATL also have tumor cells in the peripheral blood that are difficult to identify morphologically [39], but whether this model is useful in predicting the development of lymphoma-type ATL needs to be evaluated in the future.

## Conclusions

In this study, we showed an increasing trend in the PVL in HTLV-1 carriers who had a high PVL. Although our kinetic model of PVL enables the prospective prediction of the risk of developing ATL, further validation is needed to improve the accuracy of the predictions.

## Supplementary Information

Supplementary Material 1.

Supplementary Material 2.

## Data Availability

No datasets were generated or analysed during the current study.
