# Supplementary material for: Long-term kinetics of proviral load in HTLV-1 carriers: defining risk for the development of adult T-cell leukemia/lymphoma
Source: Biomark Res. 2025 Feb 26;13:34. doi: 10.1186/s40364-025-00747-5 (PMC11863581; doi:10.1186/s40364-025-00747-5)
Supplement: Supplementary file 1 — Supplementary Material 1. [file 40364_2025_747_MOESM1_ESM.pdf]

Figure S1

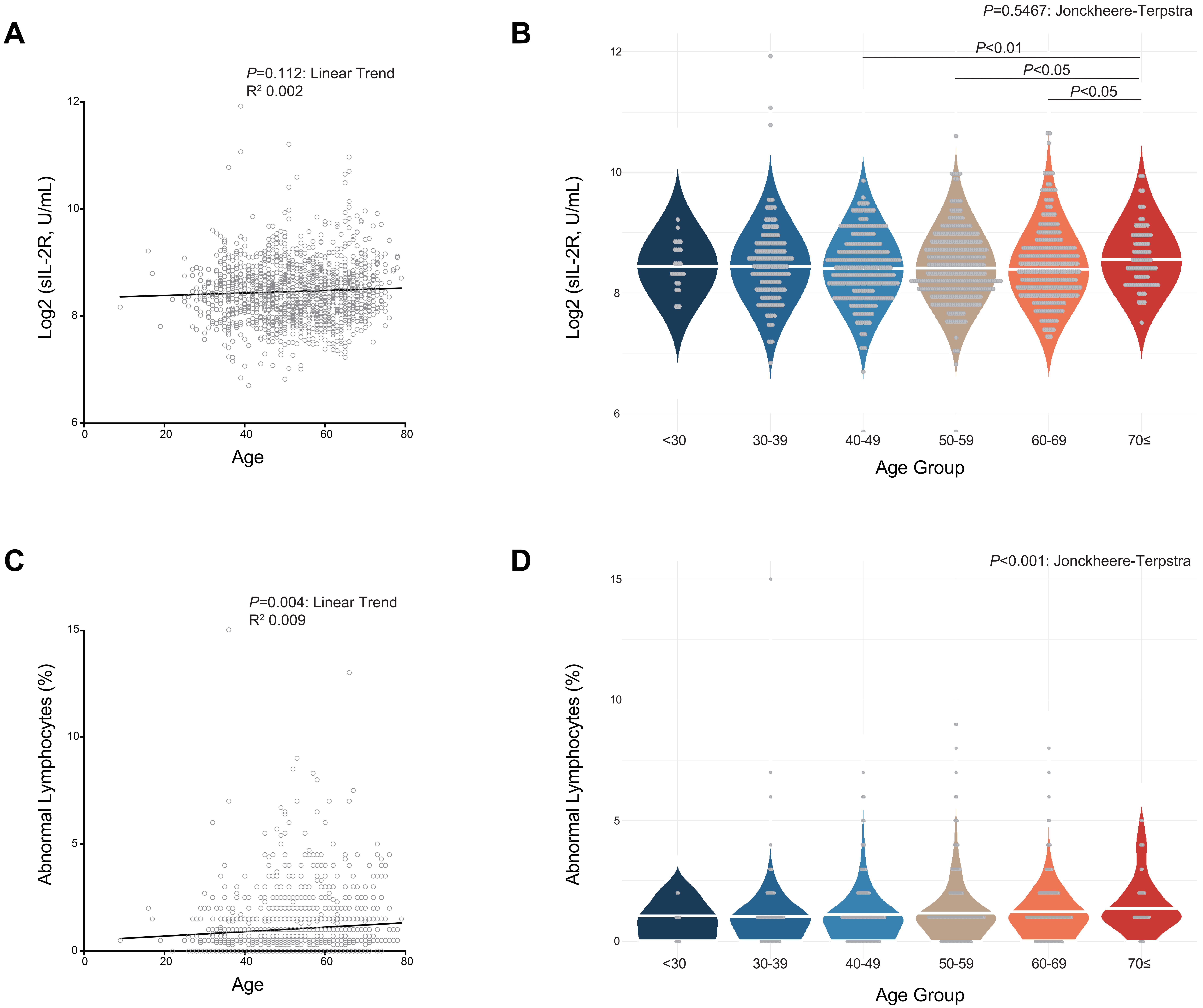

Supplementary Figure 1. Relationship between age and several parameters.
(A) Correlation between age and log-transformed soluble interleukin-2 receptor (sIL-2R) value (n=1371).
(B) Violin plots showing log-transformed sIL-2R values for each age group by 10 years (n=1371).
(C) Correlation between age and proportion of abnormal lymphocytes (n=1371).
(B) Violin plots showing proportion of abnormal lymphocytes for each age group by 10 years (n=1371).

P-values were calculated by Spearman rank correlation (A, C), Tukey-Kramer multiple test (B), or Jonckheere-Terpstra test (B, D).

Figure S2

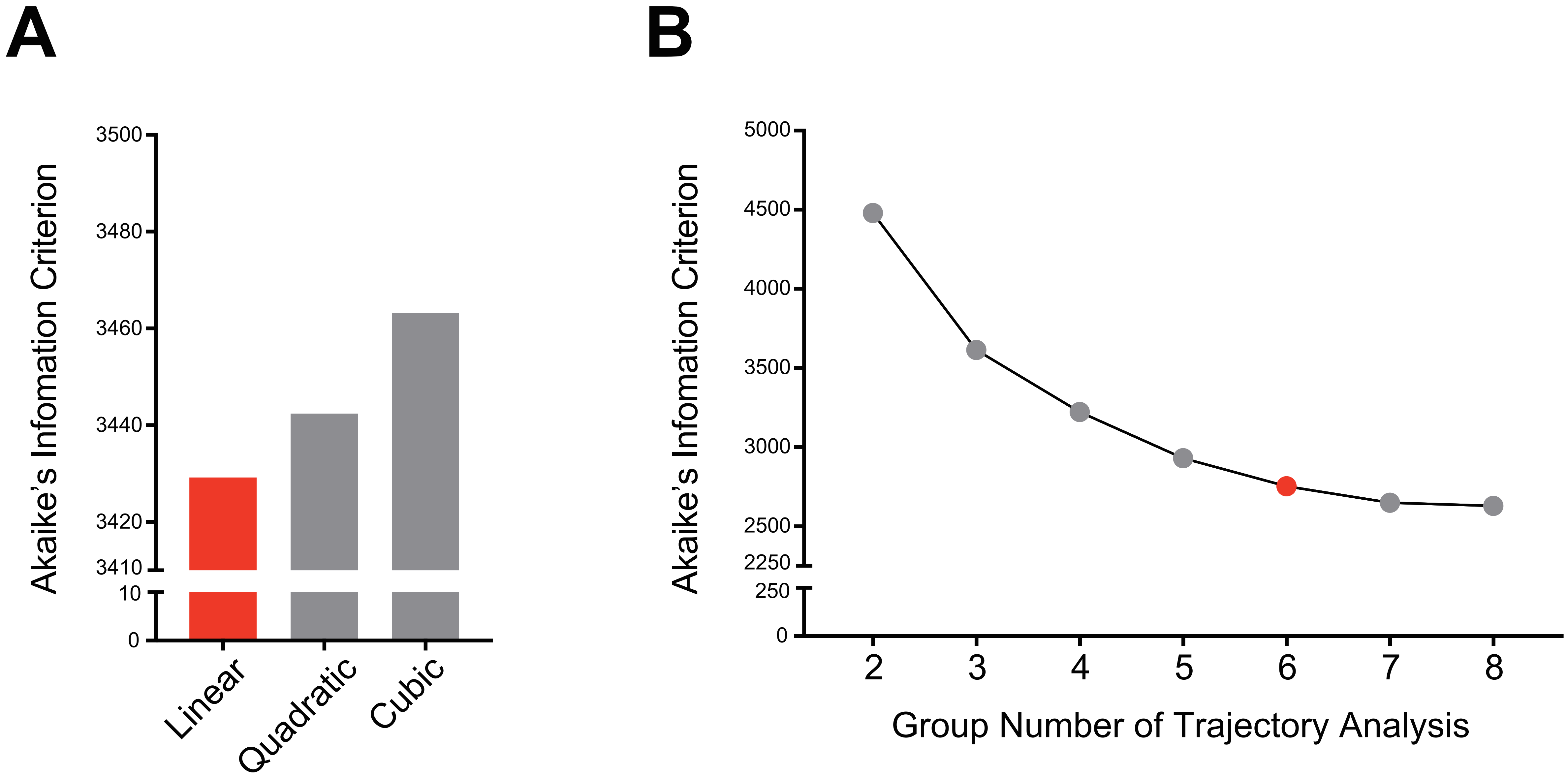

Supplementary Figure 2.

(A) Akaike's Information Criterion when linear, quadratic, and cubic functions were applied to trajectory analysis with the entire cohort as one cohort

(B) Akaike's Information Criterion from trajectory analysis when increasing the number of classes from 2 to 8.

Figure S3

61 Cases: PVL≥4% at first sample

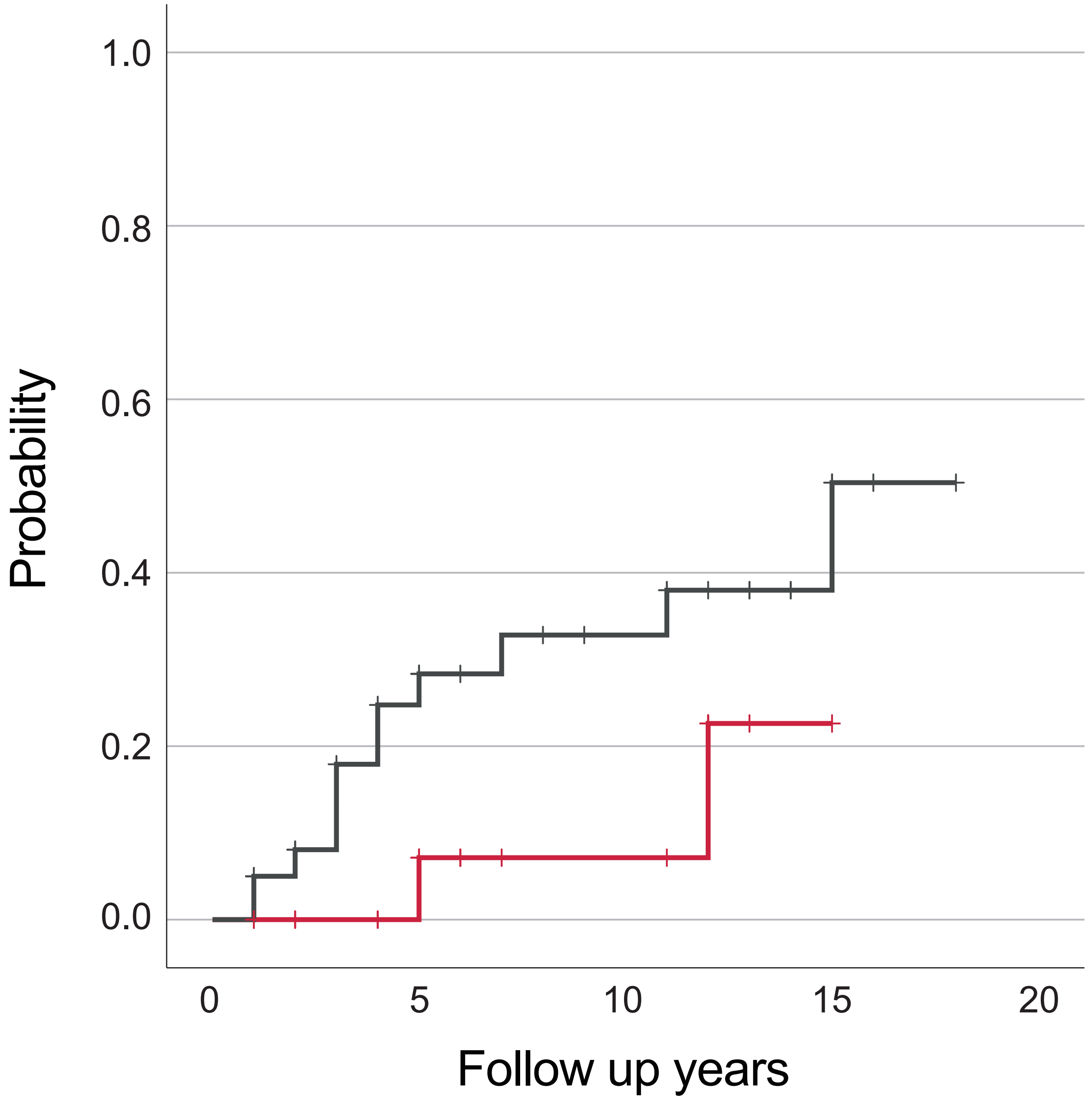

Number at risk

High	40	21	13	5	0
High-Int	21	14	7	1	0

	Development Rate of ATL, % (95% CI)	
	10 years	15 years
High	32.8% (15.6-50.0%)	50.4% (24.1-76.7%)
High-Int	7.3% (0.0-20.6%)	22.6% (7.1-52.4%)

Suppramentary Figure 3.

Cumulative incidence of ATL development according to the 2-risk groups for ATL development among 61 cases who had initial PVL values ≥4 copies/100 PBMCs.
