# Supplementary material for: Long-term kinetics of proviral load in HTLV-1 carriers: defining risk for the development of adult T-cell leukemia/lymphoma
Source: Biomark Res. 2025 Feb 26;13:34. doi: 10.1186/s40364-025-00747-5 (PMC11863581; doi:10.1186/s40364-025-00747-5)
Supplement: Supplementary file 2 — Supplementary Material 2. [file 40364_2025_747_MOESM2_ESM.docx]

Table S1. Characteristics of cases with PVL 4-8 copies/100 PBMCs at first sample.

|  | ≤55 | >55 | Total |
| --- | --- | --- | --- |
| Number of Cases | 19 | 12 | 31 |
| Risk Classification |  |  |  |
| High | 10 (53%) | 1 (8%) | 11 (35%) |
| High-Int | 9 (47%) | 11 (92%) | 20 (65%) |
| ATL Development | 3 (16%) | 1 (8%) | 4 (13%) |

Table S2. Results of applying multiple measurements to the risk classification for ATL development.

| Risk Classification | High | High-Int | Low-Int |
| --- | --- | --- | --- |
| 1st sample | 12 (80%) | 2 (13%) | 1 (7%) |
| 1st to 2nd samples | 12 (80%) | 2 (13%) | 1 (7%) |
| 1st to 5th samples* | 14 (93%) | 0 (0%) | 1 (7%) |
| 1st to ≥6th samples** | 14 (93%) | 1 (7%) | 0 (0%) |

*6 cases are the results of applying all samples (≤4 samples)

**７ cases are the results of applying all samples (≤5 samples)
